# Idiopathic Scoliosis Trends One Year After COVID-19: A Retrospective Study

**DOI:** 10.7759/cureus.32779

**Published:** 2022-12-21

**Authors:** Ana Pereira, Diana Lima, Mariana Martins, Teresa Plancha-Silva, Marta Amaral-Silva, Elsa Marques

**Affiliations:** 1 Physical Medicine and Rehabilitation, Central Lisbon University Hospital Center, Lisboa, PRT; 2 Physical Medicine and Rehabilitation, Central Lisbon University Hospital Center, Lisbon, PRT

**Keywords:** telemedicine, spine orthosis, treatment, covid-19, idiopathic scoliosis

## Abstract

Introduction

The reorganization of healthcare services during the COVID-19 pandemic was associated with compromised management of conditions not related to the pandemic.

Methods

A retrospective descriptive case series study was carried out that included patients followed up at the Spine Deformities (SD) consultation at Centro Hospitalar e Universitário Lisboa Central from January 2019 through December 2021 regarding diagnosis, treatment, referral, and the number of consultations performed.

Results

Referrals significantly dropped in 2020 (p<0.001). The average number of consultations per patient was found to not vary significantly in 2020 despite the reorganization of our healthcare unit due to the pandemic. 22% of the consultations were performed online. Idiopathic scoliosis (IS) was the primary diagnosis in 50% of the patients observed for the first time during the three-year time period. An increase of 18% was found in the number of IS patients that required bracing in 2021. However, late referrals, defined as the patient meeting surgical criteria at the time of initial presentation, did not increase.

Conclusion

Despite the significant decrease in primary care referrals during 2020, an overcompensation increase in referrals was not observed in 2021. However, the increase in the percentage of patients needing bracing might reflect a delayed initial presentation to the SD consultation.

## Introduction

As COVID-19 was spreading around the world at the beginning of 2020, a reorganization of the healthcare services in Portugal was projected, leading to the suspension of all non-urgent outpatient care. This, in turn, created an urgent need to address and find feasible solutions for all pending patient appointments. Even though telemedicine emerged as a complement to face-to-face appointments, there was a 16% to 35% drop in the number of hospital medical appointments and a more dramatic reduction in primary care consultations (from 33 to 76%) [1, 2]. This overall reorganization of healthcare services, prioritizing COVID-19 patients, compromised the management of health conditions unrelated to the pandemic [3-5].

The Scoliosis Research Society (SRS) defines scoliosis as a lateral curvature of the spine with a Cobb angle of 10º or higher and axial rotation recognized [6,7]. Idiopathic scoliosis (IS) accounts for 80% of all scoliosis cases and is defined as a structural curve of unknown origin, probably due to several causes [6]. IS is classified based on the age of the patient when it is first identified [6,7].

Treatment options for patients with scoliosis range from conservative approaches to operative treatment. The decision to treat is done considering the patient’s gender, growth potential, and magnitude of the curve [7]. Bracing should be considered for patients with curves between 20º and 40º and Risser 0-3. Curves greater than 40º are unlikely to respond to bracing. A surgical indication is considered when a curve is 50º or higher [8].

Early detection of scoliosis allows for non-operative therapies, which are most effective in curves of lesser magnitude. Therefore, patients can benefit from brace treatment, and even when avoiding surgery is not possible, surgical intervention can be done at the most opportune time [9-11]. A referral is considered timely when at the time of initial presentation there is no surgical indication [12].

In Centro Hospitalar e Universitário de Lisboa Central (CHULC) there is a specialized Spine Deformities (SD) consultation, intended mainly for pediatric patients with suspected or diagnosed scoliosis and/or hyperkyphosis, allowing timely diagnosis, clinical follow-up, and therapeutic guidance, with the aim of preventing the progression of deformities and the appearance of complications. This constitutes a reference in SD consultation for the Southern Region and Autonomous Regions in Portugal.

This paper aims to critically analyze the work carried out during the year 2020 regarding the consultation of SD, comparing with the years 2019 and 2021, and presents the methods used to overcome the difficulties that arose from the contingency state imposed by the pandemic.

This work is original and part of it was presented as a poster in October 2021 at the National Congress of Physical and Rehabilitation Medicine (PRM) in Portugal.

## Materials and methods

We conducted a case serial retrospective descriptive study including patients observed at the SD consultation in CHULC from January 2019 through December 2021. All patients observed for the first time during this period were included in the study and the data regarding diagnosis, referral source, and the number of consultations performed were collected. Subsequent consultations number were calculated, including the non-face-to-face consultations. Data regarding patients’ discharge was also collected.

When patients were diagnosed with IS, treatment options at the initial presentation were also registered. Treatment options were divided between observation (wait-and-see), bracing and surgical. The decision to treat was made according to the Scoliosis Research Society guidelines [8]. 

Patients without criteria for bracing or surgery were examined by a PRM doctor specializing in SD in a frequency determined by the risk of curve progression (considering the magnitude of the curve, growth potential, and family history, among other factors). Bracing was considered for all the patients that were prescribed a rigid thoracolumbosacral orthosis. Operative treatment was considered for all patients with a surgical indication, according to an SD Orthopedist.

Statistical analysis was performed using the IBM Statistical Package for Social Sciences (SPSS) version 27 (IBM Corp., Armonk, USA) software package. Categorical variables are presented as frequencies and percentages, and continuous variables as means and standard deviations (sd) or medians. Statistical analyses were performed with Pearson’s chi-square, and Kruskal-Wallis and Mann-Whitney tests. A p-value under 0.05 was considered statistically significant.

Patients were not directly studied, and their informed consent was not deemed necessary. Analysis was based on retrospective data collection without any identification of patients, thus not requiring specific ethics committee approval.

## Results

During this three-year period, a total of 367 patients were observed for the first time in the SD consultation.

In 2020, referrals significantly dropped (p<0.001), being below 75% between April and June compared to 2019. The source of referrals was stable in all years: PRM from other hospitals, followed by primary care (PC) and Orthopedics from CHULC. Other medical specialties responsible for the referrals were: Pediatrics, Pediatric Cardiology, Pediatric Surgery, Genetics, and Neurosurgery. The mean time between the referral and the appointment varied between 20.5 days (2019), 28.2 days (2020), and 25.8 days (2021). 

The number of first appointments was 140 in 2019, 86 in 2020, and 141 in 2021. In 2019 and 2021, each patient had an average of 2.1 consultations (sd=1.2), while in 2020 the average number of consultations per patient was 2.2 (sd=1.4). In 2020, of the 1065 consultations carried out, 22% were performed online. The main findings are summarized in Table 1.

**Table 1:** Characteristics of the observed patients (total consultations) *Mean ± standard deviation PRM: Physical and Rehabilitation Medicine

		2019	2020	2021	p-value
Number of patients		138	85	141	p<0.001
Referral	PRM	62	36	74	p=0.0223
	Primary Care	40	30	41	
	Orthopedics	32	18	24	
	Other	4	1	2	
Time to consult (days)*		20.5±16.9	28.2±32.8	25.8±19.0	
Diagnosis	Idiopathic Scoliosis	64	47	73	p=0.742
	Non – idiopathic Scoliosis	11	1	7	
	Asymmetry without rachis deviations	50	26	50	
	Idiopathic Hyperkyphosis	6	5	4	
	Scheurmann's disease	7	6	7	

The most common diagnosis in all years was IS (mainly in adolescents), followed by trunk asymmetry without rachis deviations and thoracic hyperkyphosis (in structured deviations, the main cause was Scheurmann's disease). 

Considering all patients observed: in 2019, 20% of the patients were discharged from the consultation, 7% missed follow-up, and 1% were referred to Orthopedics. In 2020, discharge occurred in 13% of the patients, 8% missed follow-up, and 1% were referred to Orthopedics. In 2021, 11% were discharged, 2% missed follow-up, and 1% were referred to Orthopedics.

IS represented about half of the patients (184 patients) observed for the first time in all years, and adolescents represented about 80% of all patients with IS. Findings regarding IS are summarized in Table 2. Considering treatment options for IS, it is possible to observe a slight increase in the percentage of bracing between 2019/2020 and 2021 (45%/44% and 62%, respectively), late referrals accounted for 14% in 2019, 6% in 2020, and 11% and 2021 (p=0.076).

**Table 2:** Characteristics of the observed patients (Idiopathic scoliosis consultations) *Mean ± standard deviation PRM: Physical and Rehabilitation Medicine

		2019	2020	2021	p-value
Number of patients		64	47	73	p=0.058
Referral	PRM	32	23	44	p=0.271
	Primary Care	11	9	13	
	Orthopedics	20	15	16	
	Other	1	0	0	
Time to consult (days)*		20.5±16.5	27.6±27.8	26.5±16.1	
Age of onset	Infantile	0	1	0	
	Juvenile	9	8	11	
	Adolescent	54	37	61	
	Adult	1	1	1	
Treatment	Observation	26 (40.6%)	23 (48.9%)	20 (27.4%)	p=0.076
	Brace	29 (45.3%)	21 (44.7%)	45 (61.6%)	
	Surgery	9 (14.1%)	3 (6.4%)	8 (11.0%)	

## Discussion

In 2021, healthcare utilization in the setting of SD consultation was not affected by COVID-19 restrictions, so we are now able to identify the impact of those restrictions until one year after the beginning of the pandemic. 

The decrease in the number of referrals between 2019 and 2020 is explained due to the decrease in non-urgent medical activity that was observed throughout the National Health System, especially in the months of the first wave of COVID-19 in Portugal. 

Despite the significant decrease in primary care referrals during 2020, an overcompensation increase in referrals was not observed in 2021, with the number of referrals during this year resembling the same as in 2019. Most patients referred to the consultation came from other PRM specialists (either in public hospitals or private), while PCs were responsible for about 1/3 of the referrals in all years. The percentage of referrals per specialty was stable in all years.

The proportion of late referrals was stable when comparing 2019 and 2021 and decreased in 2020. In this consultation, late referrals have represented 11% of patients in the last three years. This low rate of late referrals, when compared to other SD centers [13], may be explained by the sensitization done to pediatricians and primary care physicians regarding this matter and the admission criteria for the consultation that allows patients with low-magnitude scoliotic curves to be admitted in the consultation and monitored during growth potential by the SD-specialized doctors.

Regarding conservative treatment, unlike Dermott et al. [12], we observed an increase in the percentage of patients needing bracing, which might reflect a delayed initial presentation to the SD consultation. Considering that the optimal brace treatment requires early curve detection, the greater volume of candidates for bracing allows us to consider to which extent this bracing should have been done earlier.

Since the SD consultation is a reference for the Southern Region of the Country and Autonomous Regions, there were increased difficulties in face-to-face appointments due to the limitation of travel imposed during the first months of the COVID-19 pandemic.

Telemedicine during the first wave of the COVID-19 pandemic was a great improvement in consultation, allowing the monitoring of patients already enrolled in SD. Online consultation, by telephone or video call, allowed regular follow-up, whenever necessary, to request and evaluate radiological exams, and cooperation with the orthotic technician for the patient and their family/caregivers during the manufacturing and delivery of the braces. However, it was only used to keep appointments with the consultation users, as the first consultations remained face-to-face. The regular surveillance inherent to this consultation, specially designed for a pediatric population, is often associated with anxiety within the family, which gets enhanced if regular medical follow-up is not maintained. This condition has been successfully overcome with the use of telemedicine.

## Conclusions

When the diagnosis of IS is delayed, as this is a progressive condition, the treatment options can be limited and the need for surgery increases. In our cohort, the COVID-19 pandemic does not seem to have created an increase in the number of late referrals, posing instead a slight percentual increase in the number of patients needing bracing.

The maintenance of the average number of consultations per patient during the pandemic and the fact that there were no statistically significant differences in the treatment proposed to IS patients after 2020 are positive indicators regarding the work carried out during this period.
